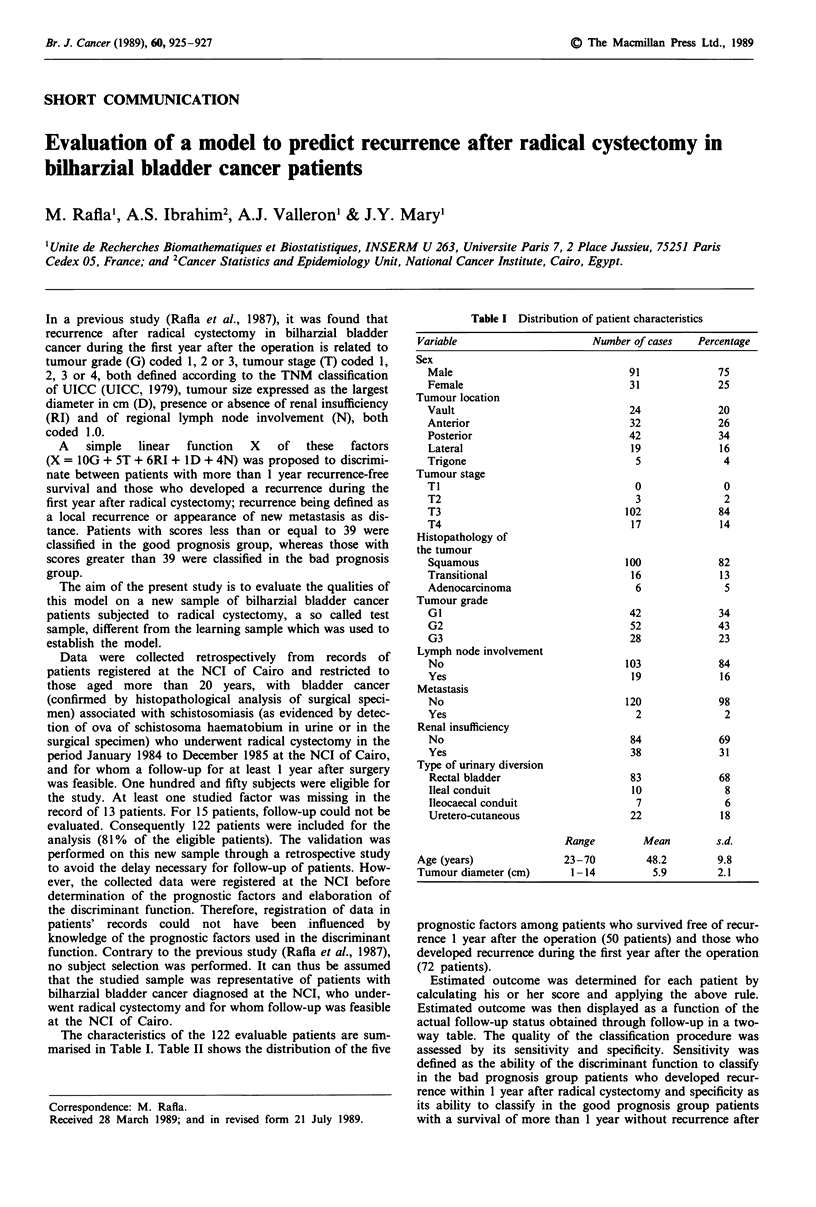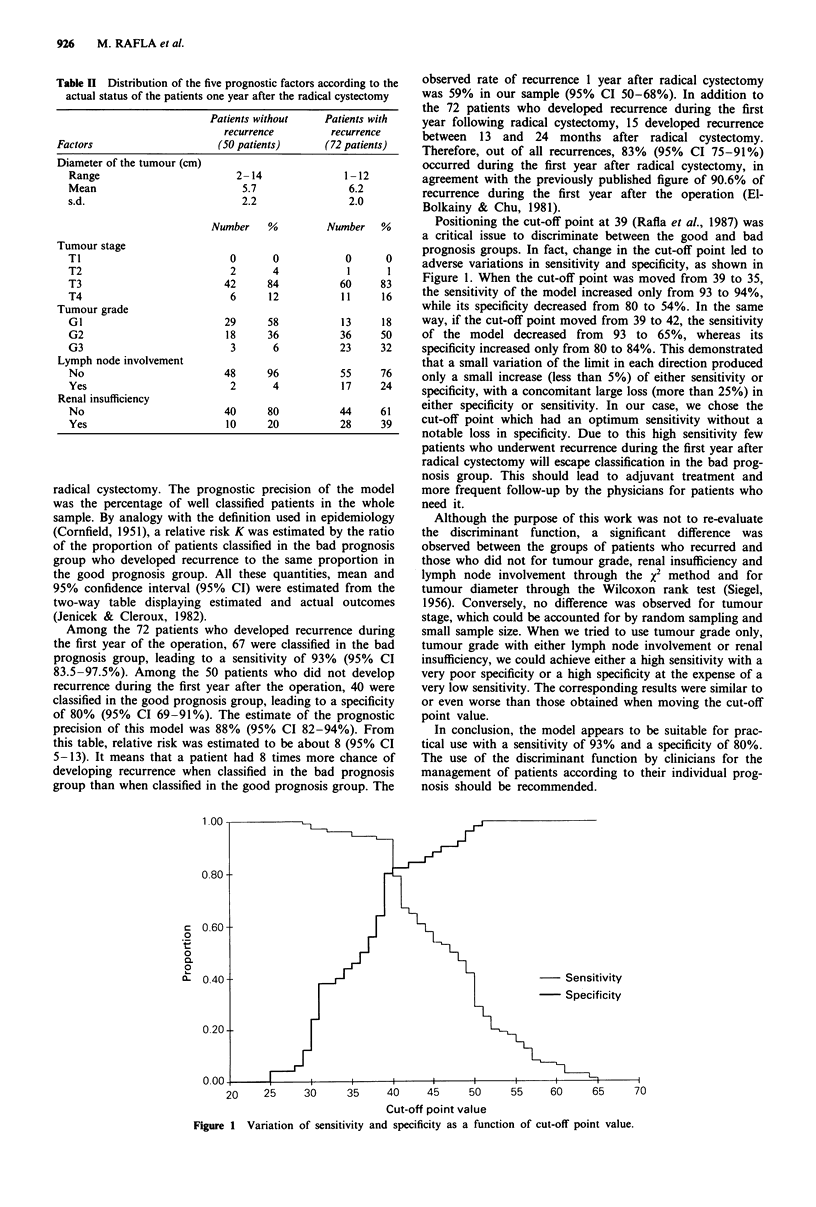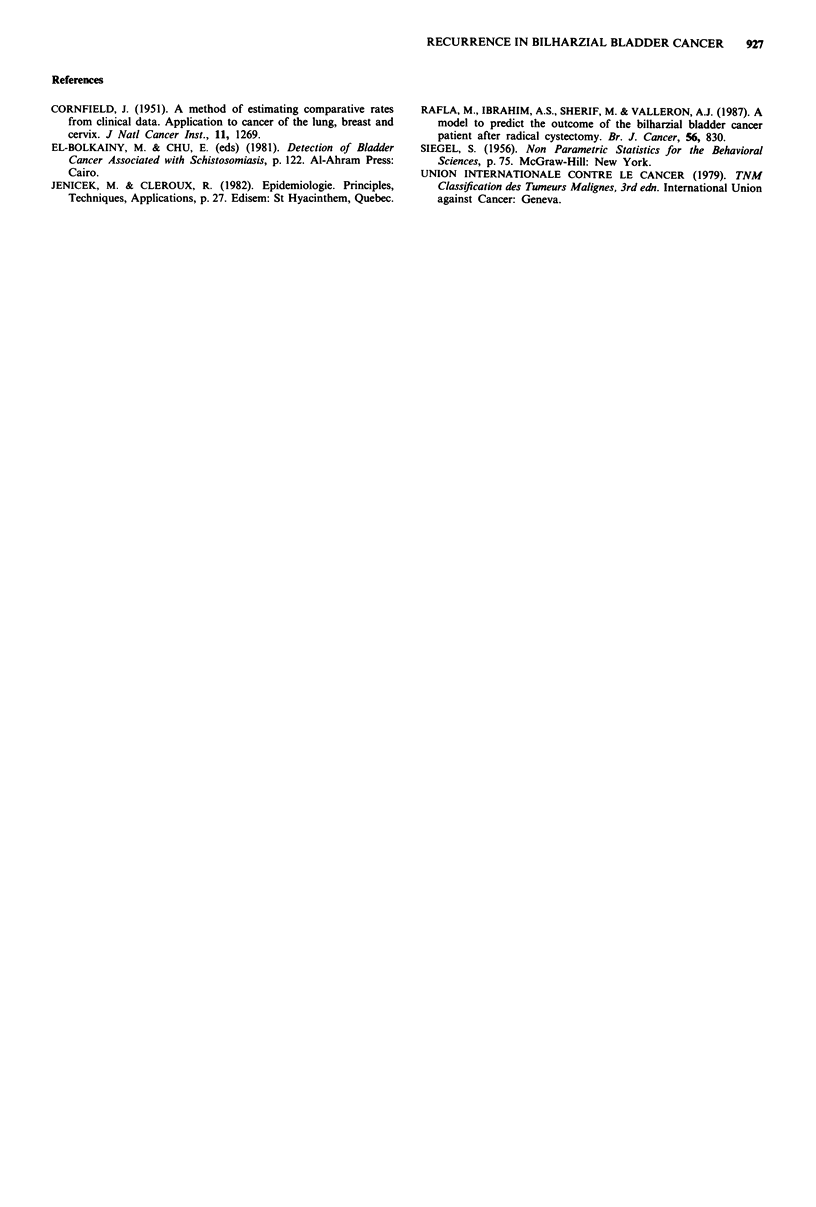# Evaluation of a model to predict recurrence after radical cystectomy in bilharzial bladder cancer patients.

**DOI:** 10.1038/bjc.1989.391

**Published:** 1989-12

**Authors:** M. Rafla, A. S. Ibrahim, A. J. Valleron, J. Y. Mary

**Affiliations:** Unite de Recherches Biomathematiques et Biostatistiques, INSERM U 263, Universite Paris, France.


Br. J. Cancer (1989), 60, 925-927 © The Macmillan Press Ltd., 1989

SHORT COMMUNICATION

### Evaluation of a model to predict recurrence after radical cystectomy in bilharzial bladder cancer patients

M. Rafla[1], A.S. Ibrahim[2], A.J. Valleron[1] & J.Y. Mary[1]

[1]*Unite de Recherches Biomathematiques et Biostatistiques, INSERM U 263, Universite Paris 7, 2 Place Jussieu, 75251 Paris Cedex 05, France; and* [2]*Cancer Statistics and Epidemiology Unit, National Cancer Institute, Cairo, Egypt.*

In a previous study (Rafla *et al.*, 1987), it was found that recurrence after radical cystectomy in bilharzial bladder cancer during the first year after the operation is related to tumour grade (G) coded 1, 2 or 3, tumour stage (T) coded 1, 2, 3 or 4, both defined according to the TNM classification of UICC (UICC, 1979), tumour size expressed as the largest diameter in cm (D), presence or absence of renal insufficiency (RI) and of regional lymph node involvement (N), both coded 1.0.

A simple linear function X of these factors ($X = 10G + 5T + 6RI + 1D + 4N$) was proposed to discriminate between patients with more than 1 year recurrence-free survival and those who developed a recurrence during the first year after radical cystectomy; recurrence being defined as a local recurrence or appearance of new metastasis as distance. Patients with scores less than or equal to 39 were classified in the good prognosis group, whereas those with scores greater than 39 were classified in the bad prognosis group.

The aim of the present study is to evaluate the qualities of this model on a new sample of bilharzial bladder cancer patients subjected to radical cystectomy, a so called test sample, different from the learning sample which was used to establish the model.

Data were collected retrospectively from records of patients registered at the NCI of Cairo and restricted to those aged more than 20 years, with bladder cancer (confirmed by histopathological analysis of surgical specimen) associated with schistosomiasis (as evidenced by detection of ova of schistosoma haematobium in urine or in the surgical specimen) who underwent radical cystectomy in the period January 1984 to December 1985 at the NCI of Cairo, and for whom a follow-up for at least 1 year after surgery was feasible. One hundred and fifty subjects were eligible for the study. At least one studied factor was missing in the record of 13 patients. For 15 patients, follow-up could not be evaluated. Consequently 122 patients were included for the analysis (81% of the eligible patients). The validation was performed on this new sample through a retrospective study to avoid the delay necessary for follow-up of patients. However, the collected data were registered at the NCI before determination of the prognostic factors and elaboration of the discriminant function. Therefore, registration of data in patients' records could not have been influenced by knowledge of the prognostic factors used in the discriminant function. Contrary to the previous study (Rafla *et al.*, 1987), no subject selection was performed. It can thus be assumed that the studied sample was representative of patients with bilharzial bladder cancer diagnosed at the NCI, who underwent radical cystectomy and for whom follow-up was feasible at the NCI of Cairo.

The characteristics of the 122 evaluable patients are summarised in Table I. Table II shows the distribution of the five prognostic factors among patients who survived free of recurrence 1 year after the operation (50 patients) and those who developed recurrence during the first year after the operation (72 patients).

**Table I** Distribution of patient characteristics

| *Variable* | | *Number of cases* | *Percentage* |
|---|---|---|---|
| Sex | | | |
| Male | | 91 | 75 |
| Female | | 31 | 25 |
| Tumour location | | | |
| Vault | | 24 | 20 |
| Anterior | | 32 | 26 |
| Posterior | | 42 | 34 |
| Lateral | | 19 | 16 |
| Trigone | | 5 | 4 |
| Tumour stage | | | |
| T1 | | 0 | 0 |
| T2 | | 3 | 2 |
| T3 | | 102 | 84 |
| T4 | | 17 | 14 |
| Histopathology of the tumour | | | |
| Squamous | | 100 | 82 |
| Transitional | | 16 | 13 |
| Adenocarcinoma | | 6 | 5 |
| Tumour grade | | | |
| G1 | | 42 | 34 |
| G2 | | 52 | 43 |
| G3 | | 28 | 23 |
| Lymph node involvement | | | |
| No | | 103 | 84 |
| Yes | | 19 | 16 |
| Metastasis | | | |
| No | | 120 | 98 |
| Yes | | 2 | 2 |
| Renal insufficiency | | | |
| No | | 84 | 69 |
| Yes | | 38 | 31 |
| Type of urinary diversion | | | |
| Rectal bladder | | 83 | 68 |
| Ileal conduit | | 10 | 8 |
| Ileocaecal conduit | | 7 | 6 |
| Uretero-cutaneous | | 22 | 18 |
| | *Range* | *Mean* | *s.d.* |
| Age (years) | 23-70 | 48.2 | 9.8 |
| Tumour diameter (cm) | 1-14 | 5.9 | 2.1 |

Estimated outcome was determined for each patient by calculating his or her score and applying the above rule. Estimated outcome was then displayed as a function of the actual follow-up status obtained through follow-up in a two-way table. The quality of the classification procedure was assessed by its sensitivity and specificity. Sensitivity was defined as the ability of the discriminant function to classify in the bad prognosis group patients who developed recurrence within 1 year after radical cystectomy and specificity as its ability to classify in the good prognosis group patients with a survival of more than 1 year without recurrence after

Correspondence: M. Rafla.
Received 28 March 1989; and in revised form 21 July 1989.

**Table II** Distribution of the five prognostic factors according to the actual status of the patients one year after the radical cystectomy

| Factors | Patients without recurrence (50 patients) | | Patients with recurrence (72 patients) | |
|---|---|---|---|---|
| Diameter of the tumour (cm) | | | | |
| Range | 2-14 | | 1-12 | |
| Mean | 5.7 | | 6.2 | |
| s.d. | 2.2 | | 2.0 | |
| | Number | % | Number | % |
| Tumour stage | | | | |
| T1 | 0 | 0 | 0 | 0 |
| T2 | 2 | 4 | 1 | 1 |
| T3 | 42 | 84 | 60 | 83 |
| T4 | 6 | 12 | 11 | 16 |
| Tumour grade | | | | |
| G1 | 29 | 58 | 13 | 18 |
| G2 | 18 | 36 | 36 | 50 |
| G3 | 3 | 6 | 23 | 32 |
| Lymph node involvement | | | | |
| No | 48 | 96 | 55 | 76 |
| Yes | 2 | 4 | 17 | 24 |
| Renal insufficiency | | | | |
| No | 40 | 80 | 44 | 61 |
| Yes | 10 | 20 | 28 | 39 |

radical cystectomy. The prognostic precision of the model was the percentage of well classified patients in the whole sample. By analogy with the definition used in epidemiology (Cornfield, 1951), a relative risk $K$ was estimated by the ratio of the proportion of patients classified in the bad prognosis group who developed recurrence to the same proportion in the good prognosis group. All these quantities, mean and 95% confidence interval (95% CI) were estimated from the two-way table displaying estimated and actual outcomes (Jenicek & Cleroux, 1982).

Among the 72 patients who developed recurrence during the first year of the operation, 67 were classified in the bad prognosis group, leading to a sensitivity of 93% (95% CI 83.5-97.5%). Among the 50 patients who did not develop recurrence during the first year after the operation, 40 were classified in the good prognosis group, leading to a specificity of 80% (95% CI 69-91%). The estimate of the prognostic precision of this model was 88% (95% CI 82-94%). From this table, relative risk was estimated to be about 8 (95% CI 5-13). It means that a patient had 8 times more chance of developing recurrence when classified in the bad prognosis group than when classified in the good prognosis group. The observed rate of recurrence 1 year after radical cystectomy was 59% in our sample (95% CI 50-68%). In addition to the 72 patients who developed recurrence during the first year following radical cystectomy, 15 developed recurrence between 13 and 24 months after radical cystectomy. Therefore, out of all recurrences, 83% (95% CI 75-91%) occurred during the first year after radical cystectomy, in agreement with the previously published figure of 90.6% of recurrence during the first year after the operation (El-Bolkainy & Chu, 1981).

Positioning the cut-off point at 39 (Rafla et al., 1987) was a critical issue to discriminate between the good and bad prognosis groups. In fact, change in the cut-off point led to adverse variations in sensitivity and specificity, as shown in Figure 1. When the cut-off point was moved from 39 to 35, the sensitivity of the model increased only from 93 to 94%, while its specificity decreased from 80 to 54%. In the same way, if the cut-off point moved from 39 to 42, the sensitivity of the model decreased from 93 to 65%, whereas its specificity increased only from 80 to 84%. This demonstrated that a small variation of the limit in each direction produced only a small increase (less than 5%) of either sensitivity or specificity, with a concomitant large loss (more than 25%) in either specificity or sensitivity. In our case, we chose the cut-off point which had an optimum sensitivity without a notable loss in specificity. Due to this high sensitivity few patients who underwent recurrence during the first year after radical cystectomy will escape classification in the bad prognosis group. This should lead to adjuvant treatment and more frequent follow-up by the physicians for patients who need it.

Although the purpose of this work was not to re-evaluate the discriminant function, a significant difference was observed between the groups of patients who recurred and those who did not for tumour grade, renal insufficiency and lymph node involvement through the $\chi^2$ method and for tumour diameter through the Wilcoxon rank test (Siegel, 1956). Conversely, no difference was observed for tumour stage, which could be accounted for by random sampling and small sample size. When we tried to use tumour grade only, tumour grade with either lymph node involvement or renal insufficiency, we could achieve either a high sensitivity with a very poor specificity or a high specificity at the expense of a very low sensitivity. The corresponding results were similar to or even worse than those obtained when moving the cut-off point value.

In conclusion, the model appears to be suitable for practical use with a sensitivity of 93% and a specificity of 80%. The use of the discriminant function by clinicians for the management of patients according to their individual prognosis should be recommended.

**Figure 1** Variation of sensitivity and specificity as a function of cut-off point value.

### References

CORNFIELD, J. (1951). A method of estimating comparative rates from clinical data. Application to cancer of the lung, breast and cervix. *J Natl Cancer Inst.*, **11**, 1269.

EL-BOLKAINY, M. & CHU, E. (eds) (1981). *Detection of Bladder Cancer Associated with Schistosomiasis*, p. 122. Al-Ahram Press: Cairo.

JENICEK, M. & CLEROUX, R. (1982). Epidemiologie. Principles, Techniques, Applications, p. 27. Edisem: St Hyacinthem, Quebec.

RAFLA, M., IBRAHIM, A.S., SHERIF, M. & VALLERON, A.J. (1987). A model to predict the outcome of the bilharzial bladder cancer patient after radical cystectomy. *Br. J. Cancer*, **56**, 830.

SIEGEL, S. (1956). *Non Parametric Statistics for the Behavioral Sciences*, p. 75. McGraw-Hill: New York.

UNION INTERNATIONALE CONTRE LE CANCER (1979). *TNM Classification des Tumeurs Malignes, 3rd edn*. International Union against Cancer: Geneva.